# Antibiotic resistance characteristics, molecular typing, and potential transmission reservoirs of methicillin-resistant *Staphylococcus aureus* in patients with postoperative infection

**DOI:** 10.3389/fmicb.2026.1630249

**Published:** 2026-03-13

**Authors:** Nan Cao, Ming-Xin Li, Ming-Yan Zhu, Yu Wang, Bing Wan, Jia Xu

**Affiliations:** 1 Department of Clinical Laboratory, Central Hospital Affiliated to Shenyang Medical College, Shenyang, China; 2 Key Laboratory of Environmental Pollution and Microecology of Liaoning Province, Clinical Experimental Teaching Center, Shenyang Medical College, Shenyang, China; 3 Clinical Practice Teaching Center, Department of Medical Microbiology, Key Laboratory of Environmental Pollution and Microecology of Liaoning Province, Shenyang Medical College, Shenyang, China

**Keywords:** antibiotic resistance, hand and foot surgery, hospital-acquired infection, methicillin-resistant *Staphylococcus aureus*, molecular typing, MRSA, *Staphylococcus aureus*

## Abstract

**Objective:**

The study aimed to assess the antibiotic resistance characteristics and molecular typing of methicillin-resistant *Staphylococcus aureus* (MRSA) in patients with postoperative infection, as well as potential MRSA reservoirs within the Hand and Foot Surgery department.

**Methods:**

Infectious specimens were collected from patients with postoperative infection in the Department of Hand and Foot Surgery at a hospital in Shenyang, China between June 2021 and June 2022. Nasal swab samples were obtained from healthcare workers, patients’ family members, and cleaning staff, and environmental samples were collected from hospital wards. Bacterial identification was performed using a mass spectrometer, and antibiotic susceptibility testing was conducted using the VITEK2-Compact automated bacterial analyzer. Molecular typing was performed *via* staphylococcal protein A (*spa*) typing and multilocus sequence typing (MLST).

**Results:**

Among 1,161 nasal swab samples, 25 MRSA strains were detected, giving a detection rate of 2.24%. One MRSA isolate was found in 77 environmental samples. Twenty-nine MRSA strains were identified in 406 postoperative infectious specimens, primarily from wound secretions and pus. Nasal swab-derived MRSA exhibited 100% resistance to benzylpenicillin and oxacillin; greater than 80% resistance to erythromycin and clindamycin; and 100% sensitivity to vancomycin, ceftaroline, and tigecycline. MRSA from infectious specimens exhibited a resistance rate of 86.21% to erythromycin and clindamycin. *spa* typing detected t437, t034, and t078 as the dominant genotypes, as they accounted for 16.36, 12.73, and 14.55% of isolates, respectively. MLST typing identified ST398, ST25, and ST59 as the predominant sequence types, each representing 19.23, 19.23, and 17.31% of isolates, respectively.

**Conclusion:**

This study identified the molecular typing, antibiotic resistance characteristics, and clonal overlaps suggesting potential transmission reservoirs of MRSA in the Department of Hand and Foot Surgery, providing important guidance for clinical treatment and hospital infection control.

## Introduction

*Staphylococcus aureus* is a common commensal and opportunistic pathogen that readily colonizes skin and mucosal surfaces, especially the anterior nares (Cheung et al., 2021). When skin or mucosal barriers are breached during surgery, the organism can invade underlying tissues and cause postoperative wound infections that prolong hospital stay and increase medical costs (Troeman et al., 2023). Methicillin-resistant *S. aureus* (MRSA) strains, which carry *mecA* or *mecC*, are resistant to all beta-lactam antibiotics, excluding fifth-generation cephalosporins such as ceftaroline (tested in this study) and ceftobiprole. In addition, they frequently exhibit multidrug resistance, making these infections difficult to treat (Hou et al., 2023a). The emergence of MRSA and antimicrobial resistance (AMR) has been a major concern in community and healthcare settings in recent decades.

Although the molecular epidemiology of MRSA has been well described globally, clonal distributions differ markedly between regions and clinical settings (Lynch and Zhanel, 2023). In China, ST239 has historically dominated hospital-associated MRSA, but recent surveillance indicates a shift toward community-associated clones such as ST59 and ST398 (Wang, 2022). The Department of Hand and Foot Surgery, characterized by open wounds and prolonged wound care, represents a high-risk microenvironment for MRSA acquisition; however, data on the current clonal landscape in this specific department are scarce.

Therefore, this study aimed to characterize the AMR profiles and determine the molecular epidemiology of MRSA isolates recovered from patients with postoperative infections, nasal swabs from healthcare workers and family caregivers, and environmental surfaces in the Department of Hand and Foot Surgery of a tertiary hospital in Shenyang. By combining *spa* typing and multilocus sequence typing (MLST), we sought to elucidate potential MRSA reservoirs, providing evidence-based guidance for targeted infection-control measures.

## Materials and methods

### Study population and bacterial strain collection

This was a prospective study conducted between June 2021 and June 2022. During each sampling round, all hospitalized patients, on-duty healthcare workers (physicians, nurses, cleaning staff), and active family caregivers present in the target wards were sampled. Postoperative infection isolates represented all microbiologically confirmed MRSA cases during the study period. Environmental samples were obtained from high-touch surfaces. This approach allowed systematic assessment of MRSA colonization reservoirs and their potential relationship with postoperative infections.

Nasal swab samples were collected from doctors, nurses, cleaning staff, and patients and their caregivers at the Central Hospital Affiliated to Shenyang Medical College between June 2021 and June 2022. Environmental samples were also obtained from frequently touched surfaces, including monitor buttons, keyboard keys, door handles, wheelchair handles, restroom handles, corridor handrails, chairs, electrocardiogram buttons, windowsills, and curtains. Additionally, all postoperative infectious specimens (including wound exudate, pus, exudate, necrotic tissue, drainage fluid) from hospitalized patients in the Departments of Hand Surgery and Foot and Ankle Surgery were collected during the same period. Postoperative infection was defined according to standard surgical site infection criteria, including local signs (erythema, purulence, or dehiscence) and/or positive microbiological culture. All samples were stored at −80 °C prior to processing. Only basic patient information was recorded, and patient identities were anonymized using numerical codes to protect privacy. This study was approved by the Ethics Committee of Shenyang Medical College (Approval No.: SYMC-20220704-003) and adhered to the principles outlined in the Declaration of Helsinki. Written informed consent was obtained from all participants.

The hospital had five Hand Surgery wards and two Foot and Ankle Surgery wards. A periodic sampling strategy was employed: two wards were selected each month for sample collection, and sampling was suspended for 2 months after all seven wards had completed one full round of sampling. This interval effectively covered the average length of hospital stay, ensuring that most patients had been discharged and new patients had been admitted before the next sampling cycle began, thereby enabling dynamic assessment of microbial community changes within the wards. In total, 15 sampling rounds (I–XV) were completed during the study period. Participants were coded as follows: hospitalized patients, physicians, nurses, cleaning personnel, and family caregivers.

(1) Collection of nasal swab samples from hospitalized patients: During each sampling round, nasal swabs were collected from all hospitalized patients in the target wards, with a particular focus on those collected from 48 h after surgery until discharge. This approach allowed monitoring of temporal changes in nasal colonization and its potential association with postoperative infections.
(2) Collection of nasal swab samples from healthcare workers: On the sampling day, sample collection was uniformly scheduled after the morning shift handover. During this period, healthcare personnel were consistently present, ensuring that nasal swabs were obtained from all on-duty staff, including doctors, nurses, and cleaning staff, thereby maintaining sample representativeness and completeness.
(3) Collection of nasal swab samples from caregivers: Caregivers included patients’ family members and hired nursing aides who provided postoperative daily care, such as repositioning, washing, and bathing assistance. Because nursing aides typically cared for multiple patients, including those located in different wards, their movement between rooms could have served as a potential mechanical vector for transmitting antimicrobial-resistant bacteria. Therefore, including caregivers in the sampling process was crucial for identifying possible cross-ward potential transmission reservoirs within the hospital.
(4) Collection of environmental samples: During each sampling round, environmental samples were collected from 14 high-touch sites in each ward (e.g., door handles, keyboards, windowsill, wheelchairs, curtain).

The inclusion of multiple sample sources was intended to enable the reconstruction of in-hospital MRSA transmission networks and to identify potential reservoirs contributing to postoperative infections, including caregivers, cleaning staff, and environmental surfaces.

#### Grouping of MRSA strains

After removing duplicate isolates, MRSA strains were categorized into two groups: nasopharyngeal swab group and postoperative infection group.

#### Matrix-assisted laser desorption/ionization time-of-flight mass spectrometry (MALDI-TOF MS) for bacterial identification

In the MALDI-TOF MS identification section, we described the stepwise sample preparation and identification process using the M-Discover 100 Excellence system (Meihua Medical, China). Reagents such as M-Discover-specific lysis buffer were included within the description of bacterial sample processing.

#### MRSA screening using the VITEK 2 system

In the MRSA screening by VITEK 2 system section, the workflow is now clearly organized to include bacterial suspension preparation (with turbidity adjusted to 0.5 McFarland using 0.85% NaCl and a turbidimeter), followed by automated identification and antimicrobial susceptibility testing using the VITEK 2-compact system and GP67 cards. MRSA determination was based on bacterial growth in cefoxitin-containing wells or the minimum inhibitory concentration (MIC) of oxacillin, interpreted according to the guidelines of the Clinical and Laboratory Standards Institute (CLSI).

#### Bacterial revival, isolation, and identification

Fresh nasal swab samples were immediately inoculated into enrichment broth and incubated for 24 h, followed by streaking onto blood agar plates. Plates were incubated in a CO₂ incubator for 24–36 h to allow colony formation. A single colony was then subcultured on a fresh blood agar plate and incubated for another 24–36 h to ensure pure MRSA isolation. Identified colonies were confirmed using the M-Discover 100 Excellence mass spectrometer according to the manufacturer’s standard operating procedures. A single colony was also used to prepare bacterial suspensions of appropriate concentration for antimicrobial susceptibility testing, and revived strains were preserved using glycerol.

Postoperative infectious specimens were retrieved from −80 °C storage for revival. These specimens were previously confirmed as MRSA during initial patient testing, and therefore did not require mass spectrometry identification or additional antimicrobial susceptibility testing. Revived strains were directly recorded and preserved using glycerol for downstream analyses.

#### Antimicrobial susceptibility testing

A tube containing 2 mL of 0.85% saline was placed in a turbidimeter for calibration. A single *S. aureus* colony was transferred to the saline solution, mixed thoroughly, and adjusted to 0.5 McFarland standard. The prepared bacterial suspension was tested using the VITEK2-Compact system with AST-GP67 antimicrobial susceptibility cards. Results were interpreted as susceptible, intermediate, or resistant according to the Clinical and Laboratory Standards Institute 2020 guidelines [Clinical and Laboratory Standard Institute (CLSI), 2020].

Following a 12-h incubation, susceptibility results were recorded. The classification of MRSA was based on cefoxitin agar growth or a minimum inhibitory concentration of oxacillin exceeding 4 μg/mL. Strains with cefoxitin-induced resistance or oxacillin resistance were classified as MRSA.

Antimicrobial susceptibility testing included 16 antibiotics: cefoxitin, penicillin, oxacillin, ceftaroline, erythromycin, clindamycin, gentamicin, levofloxacin, moxifloxacin, linezolid, daptomycin, teicoplanin, vancomycin, tigecycline, rifampin, and trimethoprim–sulfamethoxazole. In total, 117 *S. aureus* isolates were subjected to susceptibility testing. Tigecycline susceptibility was interpreted according to FDA breakpoints because of the absence of formal CLSI breakpoints. *S. aureus* ATCC 25923 and ATCC 29213 were used as quality control strains.

#### Screening for *mecA*

All MRSA isolates were confirmed through *mecA* gene typing. After PCR amplification, all products were validated by agarose gel electrophoresis. To ensure the accuracy and reliability of the typing results, strict quality control standards were implemented. First, samples with failed amplification or no amplification bands were excluded. Second, for three samples with negative sequencing results, we repeated the experiment, but the results remained invalid. This might have been attributable to bacterial DNA degradation or low concentration in the samples. To avoid misjudgment, these samples were excluded. Third, for samples with sequencing profiles exhibited peak clusters (mixed peaks), which indicated possible nonspecific primer binding or mixed templates, these were also excluded to ensure the specificity of the results.

#### Molecular typing

To provide insights into the genetic diversity and epidemiological characteristics of the MRSA strains, all isolates were subjected to staphylococcal protein A (*spa*) typing and MLST (Kumar et al., 2021) (Shanghai Sangon Biotech, Shanghai, China).

For *spa* typing, genomic DNA was extracted using a commercial bacterial DNA extraction kit following the manufacturer’s protocol. The *spa* gene was amplified by polymerase chain reaction (PCR) using specific primers, and the PCR products were sequenced. The resulting sequences were analyzed using Ridom StaphType software and compared to the *spa* database to assign *spa* types.

MLST was performed by amplifying and sequencing seven housekeeping genes (arcC, aroE, glpF, gmk, pta, tpi, and yqiL). Each locus was assigned an allele number based on sequence variations, and the combination of alleles was used to determine the sequence type (ST) according to the MLST database. The obtained STs were used to assess genetic relationships among the MRSA isolates.

All primer sequences used in this study were obtained from the internationally recognized MLST database (PubMLST; https://pubmlst.org/), corresponding to the standard amplification primers for the seven housekeeping genes of *S. aureus* (*arcC*, *aroE*, *glpF*, *gmk*, *pta*, *tpi*, and *yqiL*). The primer sequences and expected amplicon sizes strictly followed the official MLST scheme (Enright et al., 2000).

**Table:** 

Gene	Primer name	Sequence (5′ → 3′)	Amplicon size (bp)
*arcC*	arcC-up	TTGATT CAC CAG CGC GTA TTG TC	456 bp
*arcC*	arcC-dn	AGG TAT CTG CTT CAA TCA GCG	456 bp
*aroE*	aroE-up	ATC GGA AAT CCT ATT TCA CAT TC	456 bp
*aroE*	aroE-dn	GGT GTT GTA TTA ATA ACG ATA TC	456 bp
*glpF*	glpF-up	CTA GGA ACT GCA ATC TTA ATC C	465 bp
*glpF*	glpF-dn	TGG TAA AAT CGC ATG TCC AAT TC	465 bp
*gmk*	gmk-up	ATC GTT TTA TCG GGA CCA TC	429 bp
*gmk*	gmk-dn	TCA TTA ACT ACA ACG TAA TCG TA	429 bp
*pta*	pta-up	GTT AAA ATC GTA TTA CCT GAA GG	402 bp
*pta*	pta-dn	GAC CCT TTT GTT GAA AAG CTT AA	402 bp
*tpi*	tpi-up	TCG TTC ATT CTG AAC GTC GTG AA	474 bp
*tpi*	tpi-dn	TTT GCA CCT TCT AAC AAT TGT AC	474 bp
*yqiL*	yqiL-up	CAG CAT ACA GGA CAC CTA TTG GC	516 bp
*yqiL*	yqiL-dn	CGT TGA GGA ATC GAT ACT GGA AC	516 bp

For *spa* typing, the classical primer set published by (Huang et al., 2021) was used: *spa*-1113f, 5′-TAA AGA CGA TCC TTC GGT GAG C-3′; and *spa*-1514r, 5′-CAG CAG TAG TGC CGT TTG CTT-3′.

The expected amplicon length was 300–400 bp, covering the polymorphic X-region of the *spa* gene for subsequent sequence-based typing.

### Data collection

Patient demographic and clinical data, including age, sex, department, specimen source, and collection date, were recorded. For patients with postoperative infections, additional clinical information, such as the results of antimicrobial susceptibility testing, were documented when available. Environmental sample data, including sample location and surface type, were systematically logged to assess potential transmission sources. All collected data were anonymized and stored securely to ensure confidentiality.

### Statistical analysis

Statistical analysis was performed using SPSS software (IBM, Armonk, NY, USA). Categorical variables were expressed as frequencies and percentages.

## Results

### Detection of MRSA in environmental and nasal swab samples

In total, 77 environmental samples and 1,161 nasal swabs were collected after 15 rounds of sampling. Among the 77 environmental samples, two strains of *S. aureus* were detected, including one MRSA strain (Table 1).

**Table 1:** Detection of MRSA in environmental samples.

Sample source	Samples (*n* = 77)	MRSA isolates (*n* = 1)	MRSA carriage rate (%)
Door handle	16	0	0
Curtain	6	0	0
Keyboard	18	0	0
ECG machine	1	0	0
Wheelchair	11	1	1.3
Toilet grab bar	1	0	0
Monitor buttons	1	0	0
Corridor handrail	1	0	0
Telephone	3	0	0
Elevator	1	0	0
Lock	1	0	0
Environment	7	0	0
Chair	5	0	0
Windowsill	5	0	0

Among the 1,161 nasal isolates collected, the major species identified were *S. aureus* (117 isolates), *S. epidermidis* (129 isolates), *Pseudomonas aeruginosa* (41 isolates), *Proteus* spp. (23 isolates), *S. haemolyticus* (15 isolates), and *Klebsiella pneumoniae* (five isolates). Other bacterial species were detected at extremely low frequencies, and thus, they were not listed individually because of their limited statistical relevance. The 117 *S. aureus* strains detected included 25 MRSA and 92 methicillin-sensitive *S. aureus* strains. The details of nasal swab samples are presented in Table 2.

**Table 2:** Detection of MRSA in nasal swab samples.

Characteristics	Samples (*n* = 1,161)	MRSA isolates (*n* = 25)	MRSA carriage rate (%)
Sample source, *n* (%)			
Patients	585 (47.25)	13 (52.00)	2.22
Family caregivers	411 (33.20)	7 (28.00)	1.70
Nurses	92 (7.43)	2 (8.00)	2.17
Doctors	37 (2.99)	1 (4.00)	2.70
Cleaning staff or caregivers	36 (2.91)	2 (8.00)	5.56
Sex, *n* (%)			
Male	544 (46.86)	11 (44.00)	
Female	617 (53.14)	14 (56.00)	
Age (years), *n* (%)			
0–20	21 (1.81)	0 (0)	0
21–30	105 (9.04)	3 (12.00)	2.86
31–40	228 (19.64)	8 (32.00)	3.51
41–50	210 (18.09)	1 (4.00)	0.48
51–60	303 (26.10)	5 (20.00)	1.65
≥61	294 (25.32)	8 (32.00)	2.72

### Antibiotic resistance of MRSA in nasal swabs

The resistance, intermediate resistance, and sensitivity rates of MRSA strains are listed in Table 3. MRSA strains exhibited low rates of resistance to fluoroquinolones, linezolid, teicoplanin, rifampicin, and trimethoprim–sulfamethoxazole (11–24%). However, the resistance rates for erythromycin and clindamycin exceeded 80%, and the resistance rate for benzylpenicillin was 100%.

**Table 3:** Antibiotic resistance of MRSA strains in nasal swabs and environmental samples.

Antimicrobial agents	Number of MRSA isolates	Resistant, %	Intermediate, %	Susceptible, %
Benzylpenicillin	26	100	0	0
Oxacillin	26	100	0	0
Ceftaroline	26	0	0	100
Gentamicin	26	0	3.85	96.15
Levofloxacin	26	19.23	3.85	76.92
Moxifloxacin	26	11.54	11.54	76.92
Erythromycin	26	80.77	0	19.23
Clindamycin	26	80.77	0	19.23
Linezolid	26	19.23	0	80.77
Teicoplanin	26	11.54	0	88.46
Vancomycin	26	0	0	100.00
Tigecycline	26	0	0	100.00
Rifampin	26	23.08	0	76.92
Trimethoprim–sulfamethoxazole	26	23.08	0	76.92

### Detection and antibiotic resistance of MRSA from patients with postoperative infection

Among 585 patients with nasal swabs, 406 postoperative infectious specimens were collected. Overall, 29 MRSA strains were isolated from 406 postoperative infectious specimens, including 19 from wound secretions, 3 from exudate, 1 from necrotic tissue, 5 from pus, and 1 from drainage fluid. The patients’ mean age was 54.76 ± 18.68 years (range, 14–92), and the cohort included 19 male and 10 female patients. Patients’ characteristics are presented in Table 4.

**Table 4:** Detection of MRSA from postoperative infectious specimens.

Postoperative infectious specimens	Samples (*n* = 406)	MRSA isolates (*n* = 29), *n* (%)	MRSA-positive rate (%)
Sampling site			
Wound exudate	240	19 (65.52)	7.92
Pus	16	5 (17.24)	31.25
Exudate	110	3 (10.34)	2.73
Necrotic tissue	28	1 (3.45)	3.57
Drainage fluid	12	1 (3.45)	8.33

In the infectious specimens, the resistance rates of the 29 MRSA strains for levofloxacin, moxifloxacin, and ciprofloxacin ranged from 37 to 56%. The resistance rate was 17.24% for gentamicin and rifampicin, and that for erythromycin and clindamycin was significantly higher at 86.21%. The intermediate resistance and sensitivity data of other antibiotics are presented in Table 5.

**Table 5:** Antibiotic resistance of MRSA isolates from patients with postoperative infection.

Antimicrobial agents	Number of MRSA isolates	Resistant, %	Intermediate, %	Susceptible, %
Ciprofloxacin	29	55.17	20.69	24.14
Penicillin	29	100	0	0
Oxacillin	29	100	0	0
Ceftaroline	29	0	0	100.00
Gentamicin	29	17.24	17.24	65.52
Levofloxacin	29	41.38	3.45	55.17
Moxifloxacin	29	37.93	3.45	58.62
Erythromycin	29	86.21	0	13.79
Clindamycin	29	86.21	0	13.79
Linezolid	29	0	0	100.00
Daptomycin	29	0	0	100.00
Teicoplanin	29	0	0	100.00
Vancomycin	29	0	0	100.00
Tigecycline	29	0	0	100.00
Rifampin	29	17.24	0	82.76
Trimethoprim–sulfamethoxazole	29	34.48	0	65.52

### Screening for *mecA*

*mecA* gene typing was performed on 55 MRSA isolates. Five samples tested negative for *mecA*, including three samples (IIH12, IIH22, VIIIJ9TWO) with sequencing failure, one sample (2′) with PCR amplification failure, and one sample (IIIH39) displaying F-cluster peaks in the sequencing data. Thus, *mecA* typing was successful for 50 samples (90.9%, Table 6).

**Table 6:** Results of *mecA* detection.

No.	*mecA*	No.	*mecA*	No.	*mecA*
IIJ1	+	VIIIJ9TWO	−	27	+
IIH4	+	VIIIN1	+	29	+
IIH12	−	XB9	+	30	+
IIH22	−	XIH1	+	32	+
IIH42	+	XIIwheel chair TWO	+	33	+
IIIH16	+	XIIIJ29	+	35	+
IIIH17	+	4	+	36	+
IIIB3	+	8	+	38	+
IIIH39	−	10	+	1′	+
IVN7	+	13	+	2′	−
H25	+	15	+	3′	+
J3	+	17	+	4′	+
Y5	+	18	+	5′	+
VIIH41	+	20	+	6′	+
VIIH51	+	21	+	7′	+
VIIH37	+	22	+		
VIIH47	+	23	+		
VIIJ23	+	24	+		
VIJ2	+	25	+		
IXJ1TWO	+	26	+		

### *spa* typing results

**Figure 1:**
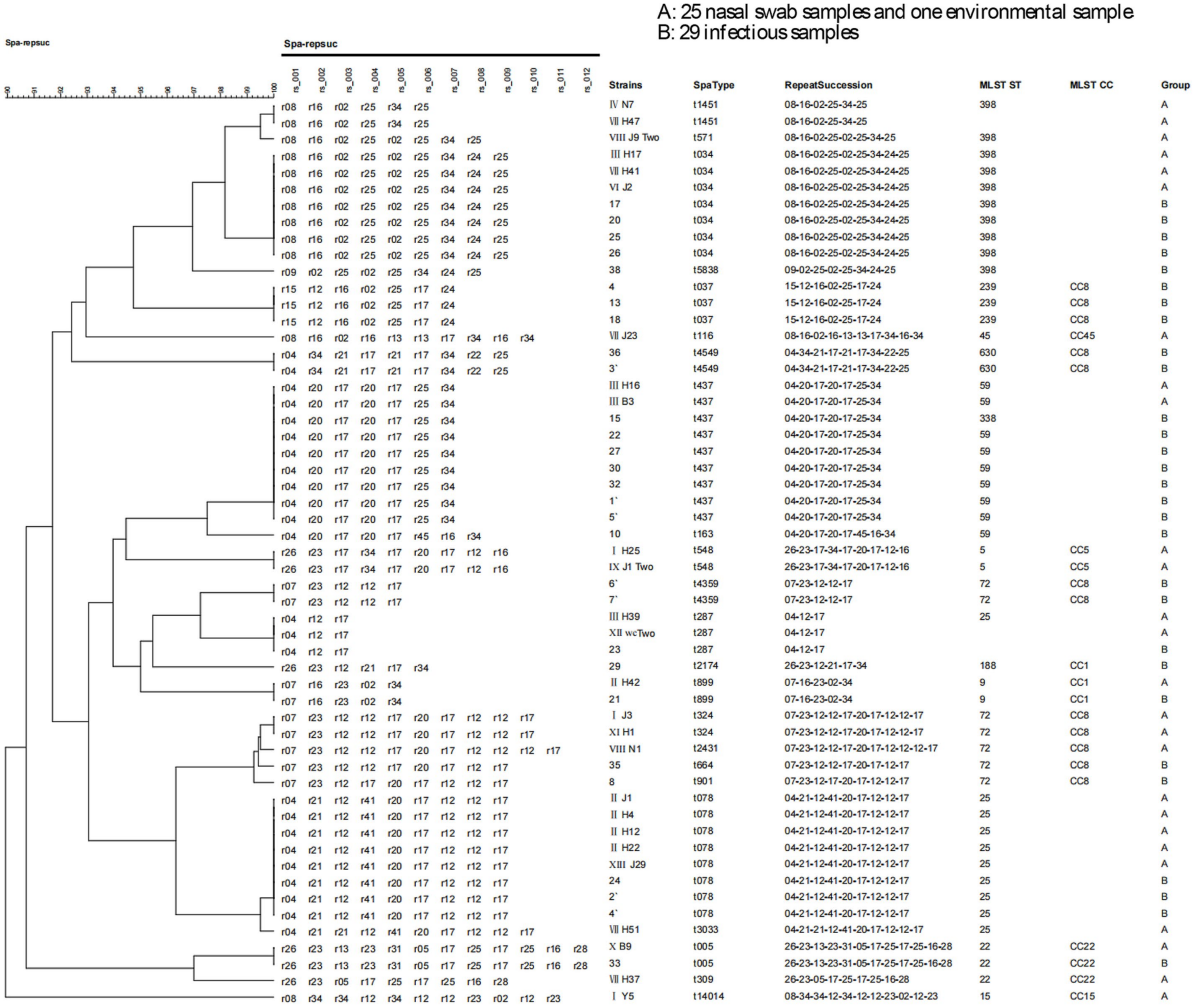
*spa* clustering tree. Fifty-five MRSA strains were subjected to *spa* typing, and 23 genotypes were identified. The details of *spa* typing results for 55 MRSA strains are presented in the *spa* clustering tree.

**Figure 2:**
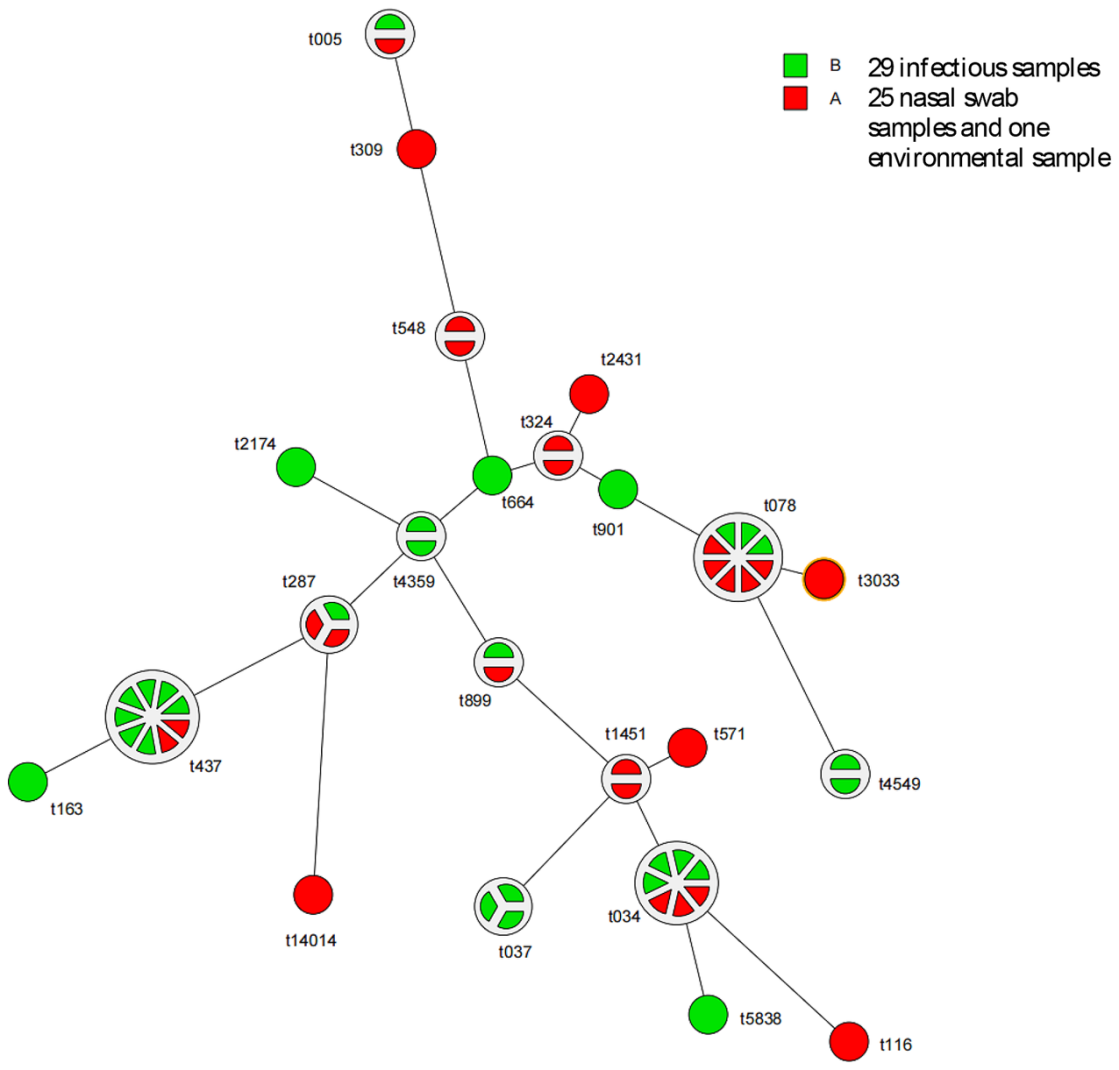
*spa* minimum spanning tree. Fifty-five MRSA strains were subjected to *spa* typing. Group A (*n* = 26) represents 25 nasal swab samples and one environmental sample, and group B represents the infectious sample group (*n* = 29), including wound secretions, pus, exudate, drainage fluid and necrotic tissue. Among 23 genotypes identified, t437, t034, and t078 were the dominant types. In the *spa* minimum spanning tree, each circle represents a *spa* type, and the area of the circle is proportional to the number of strains within that type. If the circle is divided into several sectors, then each sector corresponds to a single strain. The number next to the circle represents the *spa* type name.

Fifty-five MRSA strains were subjected to *spa* typing, and 23 genotypes were identified, with t437, t034, and t078 being the dominant types, accounting for 16.36, 12.73, and 14.55% of isolates, respectively. The *spa* typing results of 55 MRSA strains are presented in Table 7. The details of the *spa* typing results for 55 MRSA strains are presented in the *spa* clustering tree (Figure 1) and *spa* minimum spanning tree (Figure 2).

**Table 7:** *spa* typing results of 55 MRSA strains.

*spa* type	Repeat succession	MRSA isolates (*n* = 55), *n* (%)
t005	26–23–13-23-31-05-17-25-17-25-16-28	2 (3.64)
t034	08–16–02-25-02-25-34-24-25	7 (12.73)
t037	15–12–16-02-25-17-24	3 (5.45)
t078	04–21–12-41-20-17-12-12-17	8 (14.55)
t1451	08–16–02-25-34-25	2 (3.64)
t287	04–12-17	3 (5.45)
t324	07–23–12-12-17-20-17-12-12-17	2 (3.64)
t4359	07–23–12-12-17	2 (3.64)
t437	04–20–17-20-17-25-34	9 (16.36)
t4549	04–34–21-17-21-17-34-22-25	2 (3.64)
t548	26–23–17-34-17-20-17-12-16	2 (3.64)
t899	07–16–23-02-34	2 (3.64)
t571	08–16–02-25-02-25-34-25	1 (1.82)
t5838	09–02–25-02-25-34-24-25	1 (1.82)
t116	08–16–02-16-13-13-17-34-16-34	1 (1.82)
t163	04–20–17-20-17-45-16-34	1 (1.82)
t2174	26–23–12-21-17-34	1 (1.82)
t2431	07–23–12-12-17-20-17-12-12-12-17	1 (1.82)
t664	07–23–12-12-17-20-17-12-17	1 (1.82)
t901	07–23–12-17-20-17-12-12-17	1 (1.82)
t3033	04–21–21-12-41-20-17-12-12-17	1 (1.82)
t309	26–23–05-17-25-17-25-16-28	1 (1.82)
t14014	08–34–34-12-34-12-12-23-02-12-23	1 (1.82)

### MLST typing results

**Figure 3:**
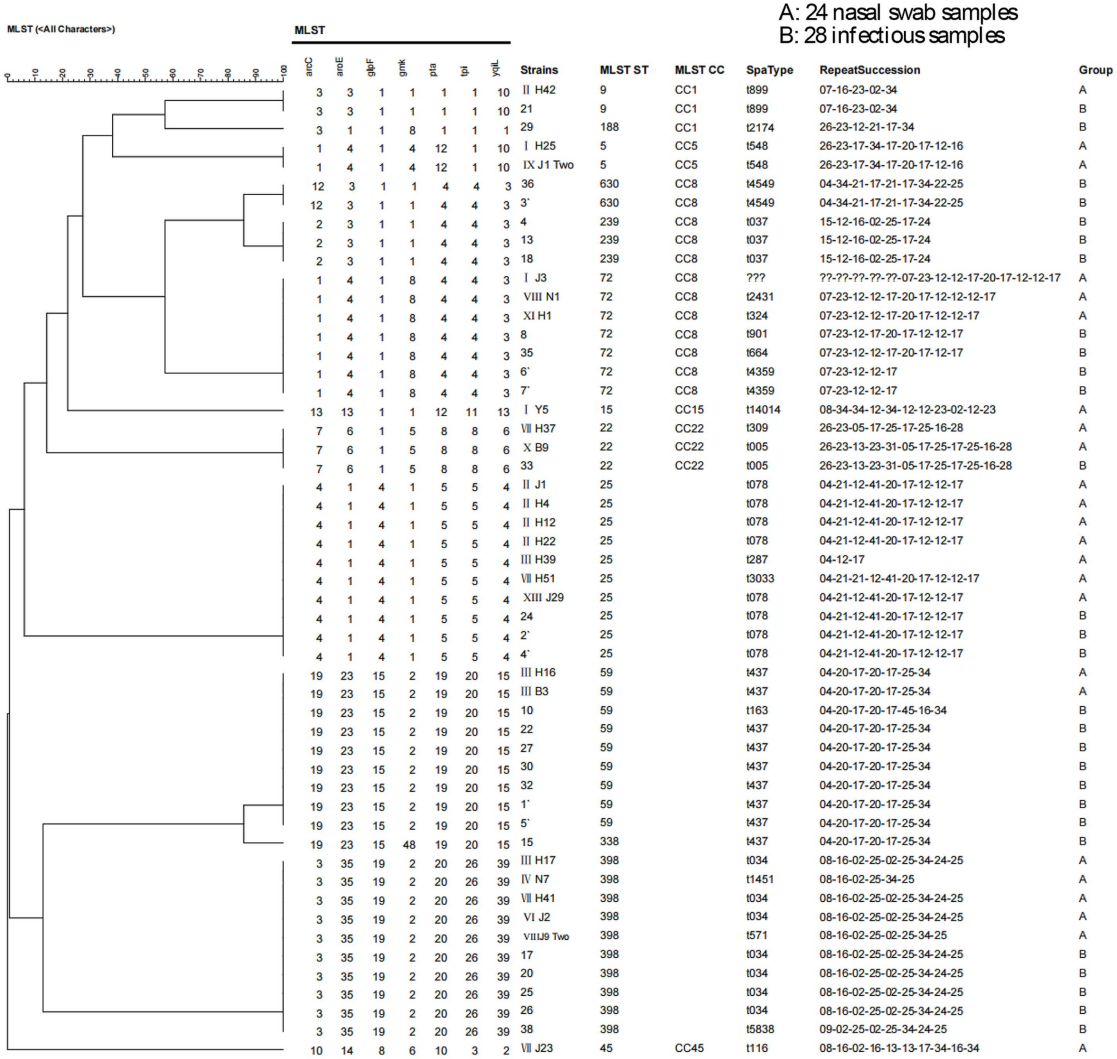
MLST clustering tree. Among the 55 MRSA strains, three samples were excluded because of failure in allele identification (one from the nasal swab group, one from the environmental sample group, and one from the infectious specimen group). In total, 52 strains were successfully typed by MLST, and the CC type was detected for some strains.

**Figure 4:**
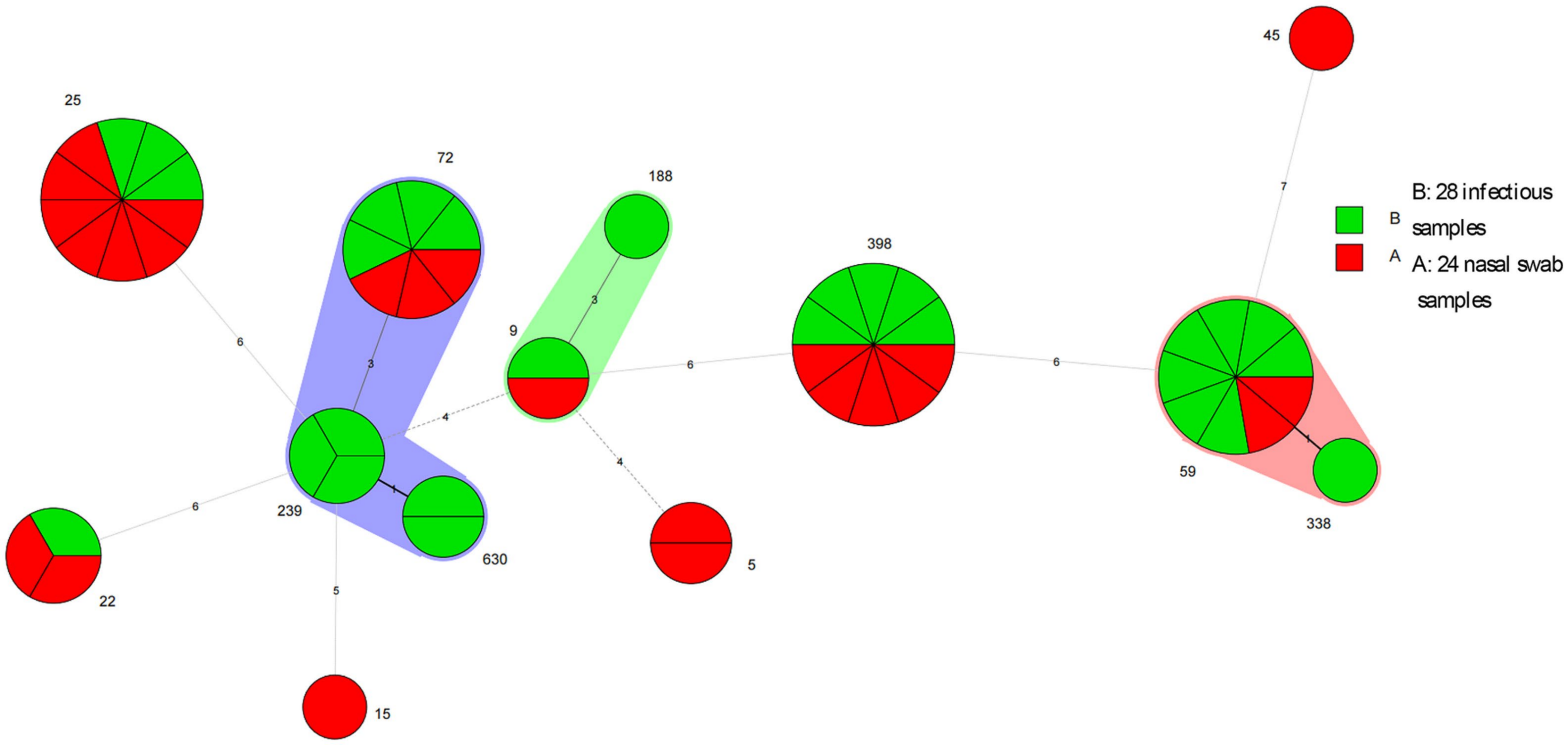
MLST minimum spanning tree. Among the 55 MRSA strains, three samples were excluded because of failure in allele identification (one from the nasal swab group, one from the environmental sample group, and one from the infectious specimen group). MLST was successful for 52 strains. Group A (*n* = 24) represents 24 nasal swab samples, and Group B (*n* = 28) represents the infectious sample group, including wound secretions, pus, exudate, drainage fluid, and necrotic tissue. The minimum spanning tree for the 52 strains is color-coded to differentiate Groups A and B. Strains within the same shading share at least four identical loci. In the minimum spanning tree, each circle represents a sequence type (ST). The size of the circle is proportional to the number of strains in that particular ST. The circle can be divided into several sections, each representing one strain within that ST. The number next to each circle indicates the corresponding ST name. The numbers along the lines connecting the circles represent the number of differing loci between the two STs. Strains within the same shading belong to the same clonal complex (CC).

Among the 55 MRSA strains, three samples were excluded because of failure in allele identification (two from the nasal swab group and one from the infectious specimen group). MLST was successful for 52 strains, and 13 genotypes were identified. Among these genotypes, ST398 and ST25 were the most prevalent, each accounting for 19.23% of isolates, followed by ST59 (17.31%) and ST72 (13.46%). Specific genotypes are detailed in Table 8, whereas the MLST clustering tree and MLST minimum spanning tree are presented in Figures 3, 4, respectively.

**Table 8:** MLST typing results of 52 MRSA strains.

ST	MLST CC	MRSA isolates (*n* = 52), *n* (%)	*arcC*	*aroE*	*glpF*	*gmk*	*pta*	*tpi*	*yqiL*
5	CC5	2 (3.85)	1	4	1	4	12	1	10
9	CC1	2 (3.85)	3	3	1	1	1	1	10
15	CC15	1 (1.92)	13	13	1	1	12	11	13
22	CC22	3 (5.77)	7	6	1	5	8	8	6
25		10 (19.23)	4	1	4	1	5	5	4
45	CC45	1 (1.92)	10	14	8	6	10	3	2
59		9 (17.31)	19	23	15	2	19	20	15
72	CC8	7 (13.46)	1	4	1	8	4	4	3
188	CC1	1 (1.92)	3	1	1	8	1	1	1
239	CC8	3 (5.77)	2	3	1	1	4	4	3
338		1 (1.92)	19	23	15	48	19	20	15
398		10 (19.23)	3	35	19	2	20	26	39
630	CC8	2 (3.85)	12	3	1	1	4	4	3

### Combination of *spa* and MLST types

Based on the molecular typing of 55 MRSA strains, three main combinations of MLST and *spa* types were observed: t078–ST25, t437–ST59, and t034–ST398. Eight MRSA isolates simultaneously exhibited the *spa* type t437 and MLST type ST59, including nasal swabs from a patient and one of the cleaning staff from the third round of sampling, as well as six specimens of wound exudate from patients with postoperative infection. Additionally, another eight strains exhibited the *spa* type t078 and MLST type ST25, including nasal swabs from three patients and one family caregiver from the second round of sampling, one family caregiver from the 13th round of sampling, and three specimens from patients with postoperative infection. Finally, seven MRSA cases simultaneously exhibited the *spa* type t034 and MLST type ST398, including nasal swabs from one patient from the third round of sampling, one family caregiver from the sixth round of sampling, one patient from the seventh round of sampling, and four specimens from patients with postoperative infection. Table 9 lists the specimen numbers from the nasal and wound secretion groups that share identical *spa* types and MLST types.

**Table 9:** Specimen numbers for the nasal swabs and wound samples that share identical *spa* and MLST types.

*spa* and MLST types	Specimen numbers of nasal swab samples	Specimen numbers of infectious samples
t005-ST22 (CC22)	XB9	33
t034-ST398	IIIH17	17
	VIIH41	20
	VIJ2	25
		26
t437-ST59	IIIH16	22
	IIIB3	27
		30
		32
		1′
		5′
t287	IIIH39	23
	XIIwheelchair two	
t899-ST9(CC1)	IIH42	21
t078-ST25	IIJ1	24
	IIH4	2′
	IIH12	4′
	IIH22	
	XIIIJ29	

## Discussion

MRSA has a broad antibiotic resistance spectrum, presenting significant challenges for clinical treatment (Wang et al., 2022). Because of the rapid mutation rate of MRSA, its molecular type varies across continents (Lynch and Zhanel, 2023). Therefore, summarizing and categorizing MRSA molecular type is crucial. From June 2021 to June 2022, the Department of Hand and Foot Surgery at the Central Hospital Affiliated to Shenyang Medical College identified three main MRSA strains: t078–ST25, t437–ST59, and t034–ST398. These findings suggest that when analyzing MRSA molecular typing, both MLST and *spa* typing should be considered to more accurately identify distinct strains (O'Hara et al., 2016; Hou et al., 2023b).

Previously, the predominant MRSA clone in most cities in China was ST239 (Wang, 2022). Recent nationwide surveillance has demonstrated a marked epidemiological shift in China, with traditional hospital-associated ST239 declining and community-associated ST59 and livestock-associated ST398 emerging in both community and healthcare settings. Our findings are consistent with this trend, as only three cases involving ST239 were identified, whereas the top three prevalent clones were ST398, ST59, and ST25. Patil et al. (2023) found that the major MRSA clone in regional hospitals of Himachal Pradesh, India was also community-associated MRSA with ST398 (39%), consistent with the predominant strain in this study. Additionally, the t437–ST59 clone identified in this study was consistent with the molecular typing results of *S. aureus* from orthopedic infections in Shanghai, China, as reported by Chen *et al.* in 2016 (Chen et al., 2016), and from various retail foods in Hangzhou, China, as studied by Chen *et al.* in 2021 (Chen et al., 2023). The ST5 clone belongs to the Japan–New York clonal lineage, which initially spread in Japan and the United States before being detected in other Asian countries (Aung et al., 2019). It has also been reported in Shenyang and Dalian. In this study, only two ST5 strains were isolated.

ST59 and ST239, two major MRSA clones, differ in virulence and resistance. ST59 carries the *chp* gene, enhancing immune evasion and virulence but displaying lower resistance, suggesting that supportive therapies might aid treatment (Chen et al., 2021a). By contrast, ST239 exhibits stronger resistance and potentially requires more potent antibiotic combinations. Among other clones, t437-ST59 is prevalent in southern China and highly resistant to clindamycin (82%) and tetracycline (41%) (Chen et al., 2021b). Its treatment is limited by resistance, and chronic infections could require prolonged courses. t548-ST5 (CC5) exhibits reduced sensitivity to vancomycin, with MICs often in the intermediate range, necessitating dosage adjustment or alternative drugs. The t899-ST9 clone, associated with livestock (especially pigs), is prevalent in Asia, and it occasionally infects farm workers, mainly causing skin and soft tissue infections. These findings highlight the risk of resistance spread attributable to antibiotic overuse in agriculture and underscore the need for cross-species monitoring. By contrast, CC1 spreads *via* human-to-human transmission, and it is linked to severe infections such as sepsis and pneumonia (Elstrom et al., 2019). Its high recombination potential increases resistance risks and complicates treatment, calling for new antimicrobial development. Thus, different MRSA clones vary regarding resistance profiles, virulence, and epidemiology, influencing treatment strategies. For example, ST239 might require broader drug combinations, whereas ST59 might respond to newer agents. Thus, antibiotic susceptibility testing and molecular typing remain essential for guiding clinical decisions.

The sources of MRSA infection can include the patients themselves, family caregivers, healthcare workers, and the hospital environment (Coia et al., 2021). In this study, the infection in patients might be linked to nasal carriage of MRSA, leading to postoperative wound infections. Moreover, family caregivers carrying MRSA in their nasal passages can also contribute to wound infections during patient care (Calderwood et al., 2023). For example, in the analysis of t078–ST25, nasal swabs from patient No. 4 (on February 25, 2022) and her family caregiver (No. 29, on February 24, 2022) obtained in the same ward within a close timeframe shared the same molecular type as the infected specimens, suggesting that caregivers could be a source of infection. Similarly, hospital cleaning staff might carry bacteria while cleaning public areas and restrooms, potentially transmitting the infection to patients (Lena et al., 2021; Alqurashi et al., 2025). In the t437–ST59 strains, nasal swabs from cleaning staff matched the molecular type of patient wound secretions, further supporting this hypothesis. Thus, the combined use of *spa* typing and MLST enabled detailed comparison of strains from different sources, helping to trace potential transmission reservoirs. For instance, identifying identical *spa*–MLST types in both nasal swabs and wound specimens suggests clonal dissemination between individuals or from the environment to patients. Conversely, differences in *spa* types among isolates with the same ST implies genetic diversity within the same lineage, potentially indicating multiple introduction events or microevolution within the hospital setting. This molecular evidence highlights the importance of surveillance among both patients and caregivers and environmental reservoirs.

In hospitals, MRSA primarily spreads through direct contact transmission (Li and Paras, 2024). Direct contact between patients, family caregivers, and healthcare workers, as well as indirect contact with hospital environments, can facilitate MRSA transmission. In this study, 25 of 1,161 nasal-swab samples (2.24%) and 1 of 77 environmental samples were positive for MRSA, indicating a low but persistent presence of the organism among individuals and on surfaces, underscoring the importance of continuous surveillance and strict hygiene measures. For instance, complex patient flow in and out of hospital wards (Sara et al., 2024), coupled with insufficient disinfection in corners, creates a risk for MRSA opportunistic infections. Additionally, if cleaning staff fail to adhere to strict hand hygiene protocols during cleaning or if hand hygiene or environmental disinfection is inadequate, cleaning staff can act as vectors for transmission, as evidenced by the detection of t437–ST59 in a cleaner’s nasal swab, matching the genotype found in six postoperative wound infections in this study. Our hospital currently implements the *Hand Hygiene Standard for Healthcare Workers* (WS/T 313–2019), which requires the use of fast-drying hand disinfectants before and after patient contact, before aseptic procedures, and after contact with the patient’s environment. Surfaces in wards and corridors and those on medical equipment are required to be wet-cleaned at least twice daily, with additional disinfection using 500 mg/L chlorine solution once daily in high-touch areas. To better control MRSA spread, regular testing and monitoring of all hospital personnel, including doctors, nurses, cleaning staff, patients, and family caregivers, are essential to promptly identify and isolate infection sources.

When treating MRSA infections, it is essential to select appropriate antibiotics based on susceptibility test results rather than relying solely on empirical treatment (Liu et al., 2022). This study found that MRSA strains in nasal swabs were resistant to penicillin and oxacillin but sensitive to vancomycin, cefalothin, tigecycline, and trimethoprim–sulfamethoxazole. Resistance rates for erythromycin and clindamycin exceeded 80%, whereas greater sensitivity was noted for linezolid, teicoplanin, and gentamicin. Strains exhibited high sensitivity to levofloxacin, moxifloxacin, rifampin, and trimethoprim–sulfamethoxazole. In addition, although vancomycin-resistant *S. aureus Staphylococcus aureus* was not detected in this study, there have been reports in China of heterogeneous vancomycin-intermediate *S. aureus*, which is vancomycin-sensitive but can produce subcolonies with varying vancomycin susceptibility on vancomycin-containing agar plates (Cheng et al., 2024). This warrants attention, highlighting the need for rational antibiotic use to delay the emergence of vancomycin-resistant MRSA. Therefore, when treating infections in patients undergoing hand and foot surgery, clinicians should refer to susceptibility results to formulate personalized treatment plans, thereby improving therapeutic outcomes and reducing the emergence of resistant strains (Nandhini et al., 2022).

Notably, the MRSA strains isolated in this study exhibited high levels of resistance to both erythromycin and clindamycin. The resistance rates among nasal swab isolates exceeded 80% for both antibiotics, whereas those from postoperative infections reached as high as 86.21%. These findings are consistent with recent multicenter surveillance data in China, suggesting that the macrolide–lincosamide–streptogramin (MLS) resistance phenotype has become widely prevalent in this region. High-level MLS resistance is primarily mediated by *erm* genes, which encode methyltransferases that modify ribosomal target sites, leading to constitutive resistance (cMLSB phenotype). As this resistance mechanism is irreversible, erythromycin and clindamycin—either alone or in combination—should be avoided in the treatment of suspected or confirmed MRSA infections, particularly in high-risk departments treating postoperative skin and soft tissue infections.

Strengthening hospital infection control is crucial for preventing and reducing MRSA infections (Coia et al., 2021). First, medical staff, especially cleaning staff, should receive continuous training in hand hygiene and disinfection protocols to ensure strict adherence. Second, educating patients and their family caregivers about MRSA infection and prevention before and after surgery is essential for raising awareness. Additionally, hospitals should regularly clean and disinfect patient rooms to reduce MRSA contamination in the environment. For high-risk populations, such as long-term hospitalized patients or immunocompromised individuals, active screening and monitoring might be necessary to detect and isolate infection sources early, thereby preventing MRSA transmission in healthcare settings (Liang et al., 2021).

This study has several limitations. First, the sample size was relatively small, with only 55 MRSA strains analyzed, which may have limited the detection of less prevalent molecular types. Second, although sampling was performed prospectively, the 1-year study period did not allow for longitudinal analysis of strain dynamics, seasonal variation, or temporal linkage between colonization and infection events. Third, molecular resolution was restricted to *spa* typing and MLST, which provide lineage-level information but do not enable high-resolution genomic comparison or inference of transmission relatedness; whole-genome sequencing (WGS) would be required to confirm clonal transmission. Finally, the study was conducted in a single specialized department (Hand and Foot Surgery) within one hospital, which may limit the generalizability of the findings to other clinical settings or geographic regions.

## Conclusion

In conclusion, this study identified three main MRSA strains based on the results of *spa* typing and MLST typing in the Department of Hand and Foot Surgery: t078–ST25, t437–ST59, and t034–ST398. Our findings demonstrated clonal overlaps between postoperative infection isolates and MRSA colonization among patients, caregivers, and healthcare workers, suggesting the presence of potential in-hospital reservoirs. The results of antibiotic resistance of MRSA strains suggest it is essential to select appropriate antibiotics based on susceptibility test results rather than relying solely on empirical treatment. These findings highlight the importance of active surveillance and targeted infection-control strategies in surgical wards.

## Data Availability

The raw data supporting the conclusions of this article will be made available by the authors, without undue reservation.

## References

[ref1] Alqurashi M. S. Sawan A. A. Berekaa M. M. Hunasemarada B. C. Al Shubbar M. D. Al Qunais A. A. . (2025). Hospital hygiene paradox: MRSA and Enterobacteriaceae colonization among cleaning staff in a tertiary Hospital in Saudi Arabia. Medicina 61:384. doi: 10.3390/medicina61030384, 40142195 PMC11944118

[ref2] Aung M. S. Urushibara N. Kawaguchiya M. Sumi A. Shinagawa M. Takahashi S. . (2019). Clonal diversity and genetic characteristics of methicillin-resistant *Staphylococcus aureus* isolates from a tertiary Care Hospital in Japan. Microb. Drug Resist. 25, 1164–1175. doi: 10.1089/mdr.2018.0468 31107152

[ref3] Calderwood M. S. Anderson D. J. Bratzler D. W. Dellinger E. P. Garcia-Houchins S. Maragakis L. L. . (2023). Strategies to prevent surgical site infections in acute-care hospitals: 2022 update. Infect. Control Hosp. Epidemiol. 44, 695–720. doi: 10.1017/ice.2023.67, 37137483 PMC10867741

[ref4] Chen X. Sun K. Dong D. Luo Q. Peng Y. Chen F. (2016). Antimicrobial resistance and molecular characteristics of nasal *Staphylococcus aureus* isolates from newly admitted inpatients. Ann. Lab. Med. 36, 250–254. doi: 10.3343/alm.2016.36.3.250, 26915614 PMC4773266

[ref5] Chen H. Yin Y. van Dorp L. Shaw L. P. Gao H. Acman M. . (2021a). Drivers of methicillin-resistant *Staphylococcus aureus* (MRSA) lineage replacement in China. Genome Med. 13:171. doi: 10.1186/s13073-021-00992-x, 34711267 PMC8555231

[ref6] Chen Q. Zhao G. Yang W. Chen F. Qi Y. Lou Z. (2023). Investigation into the prevalence of enterotoxin genes and genetic background of *Staphylococcus aureus* isolates from retain foods in Hangzhou, China. BMC Microbiol. 23:294. doi: 10.1186/s12866-023-03027-0, 37848808 PMC10580612

[ref7] Chen H. Zu Y. Luo Q. Luo X. Wang D. Li D. (2021b). Molecular epidemiological characteristics and antimicrobial susceptibility of 95 methicillin-resistant *Staphylococcus aureus* isolates from hospitalized children. J. Youjiang Med. Univ. Natl. 43, 497–502. doi: 10.3969/j.issn.1001-5817.2021.04.011

[ref8] Cheng X. Ma L. Wang Y. Sun W. Su J. (2024). Prevalence and molecular characteristics of heterogeneous vancomycin intermediate *Staphylococcus aureus* in a tertiary care center of northern China. Diagn. Microbiol. Infect. Dis. 108:116180. doi: 10.1016/j.diagmicrobio.2024.116180, 38183897

[ref9] Cheung G. Y. C. Bae J. S. Otto M. (2021). Pathogenicity and virulence of *Staphylococcus aureus*. Virulence 12, 547–569. doi: 10.1080/21505594.2021.1878688, 33522395 PMC7872022

[ref10] Clinical and Laboratory Standard Institute (CLSI) (2020). Performance standards for anti-microbial susceptibility testing. 30th Edn.

[ref11] Coia J. E. Wilson J. A. Bak A. Marsden G. L. Shimonovich M. Loveday H. P. . (2021). Joint Healthcare Infection Society (HIS) and Infection Prevention Society (IPS) guidelines for the prevention and control of meticillin-resistant *Staphylococcus aureus* (MRSA) in healthcare facilities. J. Hosp. Infect. 118, S1–S39. doi: 10.1016/j.jhin.2021.09.022, 34757174

[ref12] Elstrom P. Grontvedt C. A. Gabrielsen C. Stegger M. Angen O. Amdal S. . (2019). Livestock-associated MRSA CC1 in Norway; introduction to pig farms, zoonotic transmission, and eradication. Front. Microbiol. 10:139. doi: 10.3389/fmicb.2019.00139, 30800102 PMC6376739

[ref13] Enright M. C. Day N. P. Davies C. E. Peacock S. J. Spratt B. G. (2000). Multilocus sequence typing for characterization of methicillin-resistant and methicillin-susceptible clones of *Staphylococcus aureus*. J. Clin. Microbiol. 38, 1008–1015. doi: 10.1128/JCM.38.3.1008-1015.2000, 10698988 PMC86325

[ref14] Hou Z. Liu L. Wei J. Xu B. (2023a). Progress in the prevalence, classification and drug resistance mechanisms of methicillin-resistant *Staphylococcus aureus*. Infect Drug Resist 16, 3271–3292. doi: 10.2147/IDR.S412308, 37255882 PMC10226514

[ref15] Hou Z. Xu B. Liu L. Yan R. Zhang J. Yin J. . (2023b). Prevalence, drug resistance, molecular typing and comparative genomics analysis of MRSA strains from a tertiary a hospital in Shanxi Province, China. Front. Microbiol. 14:1273397. doi: 10.3389/fmicb.2023.1273397, 37808303 PMC10556501

[ref16] Huang J. Zhang F. Zhang J. Dai J. Rong D. Zhao M. . (2021). Molecular characterization of rifampicin-resistant *Staphylococcus aureus* isolates from retail foods in China. Antibiotics (Basel) 10:1487. doi: 10.3390/antibiotics10121487, 34943699 PMC8698944

[ref17] Kumar S. Anwer R. Yadav M. Sehrawat N. Singh M. Kumar V. (2021). Molecular typing and global epidemiology of *Staphylococcus aureus*. Curr. Pharmacol. Rep. 7, 179–186. doi: 10.1007/s40495-021-00264-7

[ref18] Lena P. Ishak A. Karageorgos S. A. Tsioutis C. (2021). Presence of methicillin-resistant *Staphylococcus aureus* (MRSA) on healthcare workers' attire: a systematic review. Trop. Med. Infect. Dis. 6:42. doi: 10.3390/tropicalmed6020042, 33807299 PMC8103237

[ref19] Li S. Paras M. L. (2024). Should contact precautions be used for patients with MRSA infection and colonization in acute care settings? NEJM Evid. 3:EVIDtt2300302. doi: 10.1056/EVIDtt2300302 38320491

[ref20] Liang B. Liang X. Gao F. Long Y. Mai J. Ai X. . (2021). Active surveillance, drug resistance, and genotypic profiling of *Staphylococcus aureus* among school-age children in China. Front. Med. 8:701494. doi: 10.3389/fmed.2021.701494, 34447764 PMC8382981

[ref21] Liu F. Rajabi S. Shi C. Afifirad G. Omidi N. Kouhsari E. . (2022). Antibacterial activity of recently approved antibiotics against methicillin-resistant *Staphylococcus aureus* (MRSA) strains: a systematic review and meta-analysis. Ann. Clin. Microbiol. Antimicrob. 21:37. doi: 10.1186/s12941-022-00529-z, 35978400 PMC9382732

[ref22] Lynch J. P. Zhanel G. G. (2023). Escalation of antimicrobial resistance among MRSA part 1: focus on global spread. Expert Rev. Anti-Infect. Ther. 21, 99–113. doi: 10.1080/14787210.2023.2154653, 36470275

[ref23] Nandhini P. Kumar P. Mickymaray S. Alothaim A. S. Somasundaram J. Rajan M. (2022). Recent developments in methicillin-resistant *Staphylococcus aureus* (MRSA) treatment: a review. Antibiotics 11:606. doi: 10.3390/antibiotics11050606, 35625250 PMC9137690

[ref24] O'Hara F. P. Suaya J. A. Ray G. T. Baxter R. Brown M. L. Mera R. M. . (2016). Spa typing and multilocus sequence typing show comparable performance in a macroepidemiologic study of *Staphylococcus aureus* in the United States. Microb. Drug Resist. 22, 88–96. doi: 10.1089/mdr.2014.0238, 26669861 PMC4722571

[ref25] Patil S. Dong S. Sharma D. Lopes B. S. Hanafiah A. Chen X. . (2023). Molecular epidemiology and characterization of multidrug-resistant MRSA ST398 and ST239 in Himachal Pradesh, India. Infect. Drug Resist. 16, 2339–2348. doi: 10.2147/IDR.S409037 37125211 PMC10134341

[ref26] Sara S. M. Thota R. C. Uddin Y. S. Bani-Yaghoub M. Sutkin G. Abourraja M. N. (2024). Patient flow modeling and simulation to study HAI incidence in an emergency department. Smart Health 32. doi: 10.1016/j.smhl.2024.100467, 38737391 PMC11085016

[ref27] Troeman D. P. R. Hazard D. Timbermont L. Malhotra-Kumar S. van Werkhoven C. H. Wolkewitz M. . (2023). Postoperative *Staphylococcus aureus* infections in patients with and without preoperative colonization. JAMA Netw. Open 6:e2339793. doi: 10.1001/jamanetworkopen.2023.39793, 37906196 PMC10618839

[ref28] Wang H. (2022). Current and future landscape of the antimicrobial resistance of nosocomial infections in China. China CDC Wkly 4, 1101–1104. doi: 10.46234/ccdcw2022.223, 36751664 PMC9889228

[ref29] Wang B. Xu Y. Zhao H. Wang X. Rao L. Guo Y. . (2022). Methicillin-resistant *Staphylococcus aureus* in China: a multicentre longitudinal study and whole-genome sequencing. Emerg Microbes Infect 11, 532–542. doi: 10.1080/22221751.2022.2032373, 35060838 PMC8843102

